# Real-Life Impact of Drug Toxicity on Dolutegravir Tolerability: Clinical Practice Data from a Multicenter Italian Cohort

**DOI:** 10.3390/v14010163

**Published:** 2022-01-17

**Authors:** Arturo Ciccullo, Gianmaria Baldin, Vanni Borghi, Filippo Lagi, Alessandra Latini, Gabriella d’Ettorre, Letizia Oreni, Paolo Fusco, Amedeo Capetti, Massimiliano Fabbiani, Andrea Giacomelli, Alessandro Grimaldi, Giordano Madeddu, Gaetana Sterrantino, Cristina Mussini, Simona Di Giambenedetto

**Affiliations:** 1 Infectious Diseases Unit, San Salvatore Hospital, 67100 L’Aquila, Italy; a.grimaldi62@gmail.com; 2 Mater Olbia Hospital, 07026 Olbia, Italy; gian.baldin@gmail.com; 3 Infectious Diseases Unit, Fondazione Policlinico Universitario Agostino Gemelli IRCCS, 00168 Rome, Italy; simona.digiambenedetto@unicatt.it; 4 Infectious and Tropical Diseases Unit, Azienda Ospedaliero Universitaria di Modena, 41125 Modena, Italy; vanni.borghi@unimore.it (V.B.); crimuss@unimore.it (C.M.); 5 Infectious and Tropical Diseases Unit, Careggi University Hospital, 50134 Florence, Italy; filippo.lagi@gmail.com (F.L.); sterrantinok@gmail.com (G.S.); 6 Infectious Dermatology and Allergology Unit, IFO S. Gallicano Institute (IRCCS), 00144 Rome, Italy; alessandra.latini@ifo.gov.it; 7 Department of Public Health and Infectious Diseases, Azienda Policlinico Umberto I, 00185 Rome, Italy; gabriella.dettorre@uniroma1.it; 8 Infectious Diseases Unit, DIBIC Luigi Sacco, University of Milan, 20157 Milan, Italy; letizia.oreni@alice.it (L.O.); andrea.giacomelli@unimi.it (A.G.); 9 Infectious and Tropical Diseases Unit, Department of Medical and Surgical Sciences, “Magna Graecia” University of Catanzaro, 88100 Catanzaro, Italy; paolofusco89@gmail.com; 10 1st Division of Infectious Diseases, Luigi Sacco University Hospital, 20157 Milan, Italy; amedeo.capetti@asst-fbf-sacco.it; 11 Infectious Diseases Unit, Azienda Ospedaliero-Universitaria Senese, 53100 Siena, Italy; massimiliano.fabbiani@gmail.com; 12 Unit of Infectious Diseases, Department of Medical, Surgical and Experimental Sciences, University of Sassari, 07100 Sassari, Italy; giordano@uniss.it; 13 Department of Safety and Bioethics, Catholic University of the Sacred Heart, 00168 Rome, Italy

**Keywords:** HIV, HAART, dolutegravir, toxicity

## Abstract

Dolutegravir (DTG) is currently one of the most used Integrase inhibitors (INI) in antiretroviral therapies (ARV) in both naïve and experienced people living with HIV (PLWHIV). We analyzed a multicenter cohort of PLWHIV, both naïve and experienced, starting an ARV including DTG. We enrolled 3775 PLWHIV: 2763 (73.2%) were males, with a median age of 50 years. During 9890.7 PYFU, we observed 930 discontinuations (9.4 per 100 PYFU). Estimated probabilities of maintaining DTG at three and five years were 75.1% and 67.2%, respectively. Treatment-naïve pts showed a lower probability of maintaining DTG at three and five years compared to treatment-experienced PLWHIV (log-rank *p* < 0.001). At a multivariate analysis, a longer time of virological suppression (aHR 0.994, *p* < 0.001) and having experienced a previous virological failure (aHR 0.788, *p* = 0.016) resulted protective against DTG discontinuation. Most discontinuations (84.0%) happened within the first 12 months of DTG initiation, in particular, 92.2% of discontinuations due to neuropsychiatric toxicity were observed in the first year. Our data confirm the overall good tolerability of DTG in clinical practice, with a low rate of discontinuations. CNS toxicity resulted the main reason for DTG discontinuation, with most related interruptions happening in the first year from DTG introduction.

## 1. Introduction

Dolutegravir (DTG), a second-generation integrase inhibitor (INI), has shown high efficacy and safety in both naïve and treatment-experienced (TE) people living with HIV (PLWHIV) [1,2], in both three-drug regimens, as well as in two-drug regimens with either lamivudine or rilpivirine [3,4]. Clinical practice studies have shown the optimal tolerability profile of DTG-based strategies [5,6]. Nevertheless, reports from clinical practice about the high rate of neuropsychiatric events in patients treated with DTG leading to treatment discontinuation (TD) [7] have raised questions on the tolerability of DTG-based regimens. The aim of this study was to evaluate the overall tolerability of DTG-based regimens in an Italian multicenter cohort of PLWHIV.

## 2. Materials and Methods

We analyzed data from a multicenter cohort [8] of adult (age ≥ 18 years) PLWHIV starting for the first time any DTG-containing regimen. We evaluated both time to virological failure (VF, defined as failure to achieve HIV-RNA < 50 copies/mL for naïve PLWHIV or experienced PLWHIV on a failing regimen after 24 weeks from treatment initiation and defined by a single HIV-1 RNA ≥ 1000 copies/mL or by two consecutive HIV-1 RNA ≥ 50 copies/mL in virologically suppressed PLWHIV) and time to TD (defined as the discontinuation of DTG regardless of whether the remaining antiretroviral drugs used in the combination had been changed or not) for any cause, using Kaplan–Meyer survival analysis. Cox regression analysis was performed to evaluate predictors of TD. We collected age, sex, risk factors for HIV infection, ARV history, peak HIV-1 RNA, CD4+ cells count nadir and viro-immunological parameters at baseline. 

## 3. Results

We enrolled 3775 PLWHIV: 2763 (73.2%) were males, with a median age of 50 years (IQR 42–56). Naïve PLWHIV were 702 (18.6%), of whom 128 (18.2%) were AIDS-presenters. As to experienced PLWHIV, median time from HIV diagnosis was 17.0 years (9.0–24.8) while median time on ARV was 13.4 years (6.7–19.9); 393 (12.8%) had an HIV-RNA > 50 cp/mL at baseline. Full population characteristics are available in Table 1.

During 9890.7 PYFU, we observed 930 discontinuations, a rate of 9.4 per 100 PYFU. The reasons for DTG discontinuation were: toxicity (34.6% of total discontinuations), simplification to a Single Tablet Regimen (STR, 17.8%), treatment intensification (17.0%), death due to non-HIV-related issues (3.7%), drug–drug interaction (1.9%), pregnancy (1.1%), concern of cardiovascular toxicity (1.1%), other/unknown (22.8%).

Regarding discontinuations due to toxicity, the most frequently reported causes were: Central Nervous System (CNS) toxicity (129 PLWHIV, 3.4% of total population), gastrointestinal (GI) toxicity (58, 1.5%), hypersensitivity (26, 0.7%), and renal toxicity (19, 0.5%). We further investigated neuropsychiatric events leading to TD. In detail, there were cases of: insomnia (34, 0.9%), headache (31, 0.8%), depression (26, 0.7%), anxiety (26, 0.7%), nightmares/hallucinations (6, 0.2%) and vertigo (6, 0.2%). PLWHIV experiencing TD due to neuropsychiatric events were predominantly males (95, 73.6%), with a median age of 49 years (IQR 40–55). No significant differences were observed in terms of baseline characteristics between PLWHIV who experienced CNS toxicity and those who did not.

Estimated probabilities of maintaining DTG at 144 and 240 weeks were 75.1% (SD ± 0.8%) and 67.2% (SD ± 0.9%), respectively. Treatment-naïve PLWHIV showed a lower probability of maintaining DTG at 144 and 240 weeks (64.6% and 57.8%, respectively) compared to treatment-experienced PLWHIV (77.4% and 69.2%, log-rank *p* < 0.001). In particular, we observed that both virologically suppressed experienced PLWHIV (aHR 0.58, 95% Confidence Interval (CI) 0.50–0.68, *p* < 0.001) and experienced PLWHIV with a detectable HIV-RNA at baseline (aHR 0.76, 95% CI 0.60–0.95, *p* = 0.016) had a lower risk of discontinuing DTG compared to naïve PLWHIV. At a multivariate analysis, a longer time of virological suppression (per 10 months more, B -0.06, aHR 0.994, 95% CI 0.992–0.996, *p* < 0.001) and having experienced a previous virological failure (B-0.238, aHR 0.788, 95% CI 0.650–0.956, *p* = 0.016) resulted protective against DTG discontinuation, after adjusting for CDC stage, peak HIV-RNA and nadir CD4+ cell count. The vast majority of discontinuations (781/930, 84.0%) happened within the first 12 months of DTG initiation. 

In our cohort, we observed 318 VF over 9499.8 PYFU, a rate of 3.3 VF per 100 PYFU. Estimated probability of not experiencing VF was 96.1% (SD ± 0.3%) at 48 weeks, 90.1% (SD ± 0.6%) at 144 weeks and 87.0% (SD ± 0.8%) at 240 weeks. In our regression analysis, we found that a peak HIV-RNA over 500,000 copies/mL (vs. lower than 500,000 copies/mL, aHR 1.86, 95% CI 1.32–2.61, *p* < 0.001) and a previous episode of VF (aHR 1.51, 95% CI 1.10–2.08, *p* = 0.012) were associated with a higher risk of failing DTG while months of virological suppression at baseline (per 10 months more B-0.15, aHR 0.98, 95% CI 0.98–0.99, *p* < 0.001) resulted protective against VF.

In a specific survival analysis, the probability of not discontinuing DTG due to neuropsychiatric toxicity was 88.5% at one year, 77.7% at three years, and 75.4% at five years of follow-up. No differences in this regard were observed between naïve or experienced pts. In our regression analysis, concomitant abacavir use was not a predictor of discontinuations due to CNS toxicity (*p* = 0.335). Discontinuations due to CNS toxicity showed an overall rate of 1.3 per 100 PYFU and they were observed almost entirely in the first year since DTG initiation (119/229, 92.2%).

## 4. Discussion

Our data confirm the good tolerability profile of DTG in a large multicenter cohort of 3775 PLWHIV. Indeed, confirming previous findings [9], in our work, we reported less than 10 TD per 100 PYFU, with over 80% of TD observed in the first year. Only a portion of TDs were due to adverse events: We reported an incidence rate of discontinuations due to all toxicities of 3.2 per 100 PYFU and, in particular, a rate of discontinuations due to neuropsychiatric events incidence rate of 1.30 per 100 PYFU. 

In our cohort, experienced PLWHIV presented a lower risk of discontinuing DTG compared to treatment-naive ones. This is highlighted both from the significant difference in terms of DTG discontinuation observed between naïve and treatment-experienced PLWHIV and from the fact that, in our regression analysis, a previous virological failure and a longer time of virological suppression (both found in heavily-experienced individuals) resulted protective against TD. These findings are similar to those observed by Penafiel et al. [10] and suggest that DTG is considered by clinicians a fundamental drug in salvage regimens.

Data from our cohort show a lower rate of neuropsychiatric events leading to TD compared to other studies [11,12,13]. In this particular instance, our analyses failed to observe any predictor of TD due to CNS toxicity, although in other studies from our cohort [14], HCV-serostatus appeared to be associated with this event. In particular, we did not observe a significant association between co-administration of abacavir and CNS toxicity, a finding reported in previous studies [13].

Regarding TD due to pregnancy, new data from the Tsepamo study re-evaluate the potential effects of DTG on neural tube development and may change clinicians’ perspective on DTG use in young women [15].

Even if our study was not specifically designed to investigate efficacy, we observed a very low rate of VF, in line with what we expected from the results of previous studies [16]. As previously observed, PLWHIV with a higher peak HIV-RNA at time of diagnosis, had a higher risk of incurring in VF [14]. 

Our study’s main limitations are its retrospective design and the fact that low-grade toxicity not requiring treatment interruption was not registered in the cohort’s database. Instead, the long follow-up time and the large sample size represent the main strengths of our work. 

In conclusion, our work confirms the high tolerability of DTG in a clinical-practice setting, both in naive and TE PLWHIV. There has been significant controversy regarding the potential role of DTG in inducing neuropsychiatric toxicity and, with our study, we tried to evaluate the “real-life” impact of DTG-based strategies on neuropsychiatric toxicity events.

## Figures and Tables

**Table 1:** Characteristics of the study population.

Variables	Overall n = 3775
Males, n (%)	2763 (73.2)
Age, Years, Median (IQR)	50.4 (41.6–56.1)
HIV Risk factors, n (%)	
Heterosexual	1447 (38.3)
MSM	1377 (36.5)
IDU	592 (15.7)
Others/Unknown	359 (9.5)
HCV Ab positive, n (%)	456 (12.1)
HBsAg positive, n (%)	97 (2.6)
CDC stage C, n (%)	932 (24.7)
Years from HIV diagnosis (for TE), Median (IQR)	17.0 (9.0–24.8)
Zenith HIV-RNA, log_10_ cp/mL, Median (IQR)	5.07 (4.53–5.53)
Nadir CD4+, cells/mmc, Median (IQR)	200 (65–332)
Reasons for starting study drug, n (%)	
Naive	702 (18.6)
Treatment failure	318 (8.4)
Simplification	1722 (45.6)
GI/hepatic toxicity	148 (3.9)
Dyslipidemia	214 (5.7)
Renal toxicity	87 (2.3)
CNS toxicity	29 (0.8)
Rash/hypersensitivity	17 (0.5)
Osteoporosis	47 (1.2)
Other toxicities	39 (1.0)
Drug–drug interactions	161 (4.3)
Restart after interruption	28 (0.7)
Cardiovascular disease	17 (0.5)
Other/Unknown	246 (6.5)
Years on ARV (for TE), Median (IQR)	13.4 (6.7–19.9)
Months of virological suppression (for TE), Median (IQR)	23.5 (5.7–96.8)
Previous virological failure (for TE), n (%)	1482 (48.2)
Virological suppressed at baseline (for TE), n (%)	2680 (87.2)
Therapies before switch (for TE), n (%)	
2NRTI + PI	915 (29.8)
2NRTI + NNRTI	763 (24.8)
2NRTI + INI	407 (13.3)
Mono/Dual	609 (19.8)
Others	379 (12.3)
Previous INI exposure (for TE), n (%)	860 (28.0)
Reasons for discontinuation, n (% of total discontinuation)	
Intensification	158 (17.0)
Simplification	166 (17.8)
GI/hepatic toxicity	58 (6.2)
Dyslipidemia	4 (0.4)
Renal toxicity	19 (2.1)
CNS toxicity	129 (13.9)
Rash/hypersensitivity	26 (2.8)
Osteoporosis	4 (0.4)
Other toxicities	82 (8.8)
Drug–drug interactions	18 (1.9)
Pregnancy	10 (1.1)
Cardiovascular disease	10 (1.1)
Death	34 (3.7)
Other/Unknown	212 (22.8)

## Data Availability

The data presented in this study are not publicly available but are available on request from the corresponding author.
